# Association of the Amount and Pattern of Physical Activity With Arterial Stiffness: The Maastricht Study

**DOI:** 10.1093/geroni/igaa057.2863

**Published:** 2020-12-16

**Authors:** Evelien Vandercappellen, Ronald Henry, Coen Stehouwer, Annemarie Koster

**Affiliations:** 1 Maastricht University Medical Center, Maastricht, Netherlands; 2 Maastricht University Medical Center+, Maastricht, Limburg, Netherlands; 3 Maastricht University, Maastricht, Limburg, Netherlands

## Abstract

We examined the associations of the amount and the pattern of higher intensity physical activity with arterial stiffness. Data from The Maastricht Study (n=1699; mean age: 60±8 years, 49.4% women, 26.9% type 2 diabetes (T2DM)) were used. Arterial stiffness was assessed by carotid-to-femoral pulse wave velocity (cfPWV). The amount (hours/day) and pattern of higher intensity physical activity were assessed with the activPAL3®. Activity groups were: inactive (<75min/week), insufficiently active (75-150min/week), weekend warrior (>150min/week in ≤2 sessions), and regularly active (>150min/week in ≥3 sessions). After full adjustment, higher intensity physical activity was associated with lower cfPWV (amount: -0.35[-0.65;-0.05], insufficiently active: -0.33[-0.55;-0.11]; weekend warrior: -0.38[-0.64;-0.12] and regularly active: -0.46[-0.71;-0.21] (reference: inactive)). These associations were stronger in those with T2DM. Participating in higher intensity physical activity was associated with lower cfPWV, regardless of the weekly pattern, and may be an important strategy to reduce CVD risk, particularly in T2DM.

